# Foot loading patterns in normal weight, overweight and obese children aged 7 to 11 years

**DOI:** 10.1186/1757-1146-6-36

**Published:** 2013-08-28

**Authors:** Stephen D Cousins, Stewart C Morrison, Wendy I Drechsler

**Affiliations:** 1 School of Health, Sport and Bioscience, University of East London, Stratford, London, England; 2 La Trobe Rural Health School, Faculty of Health Sciences, La Trobe University, Flora Hill Campus, Bendigo, Victoria, Australia

**Keywords:** Gait, Childhood obesity, Feet

## Abstract

**Background:**

Childhood obesity is thought to predispose to structural foot changes and altered foot function. Little is currently understood about whether similar changes occur in overweight children. The aim of this study was determine foot loading characteristics in obese, overweight and normal weight children aged 7 to 11 years during level walking.

**Methods:**

Dynamic plantar pressures were measured in 22 obese, 22 overweight and 56 normal weight children recruited from local primary and secondary schools in East London. Peak pressure, peak force, normalised peak force, pressure–time and force-time integrals were analysed at six regions of the plantar foot: lateral heel, medial heel, midfoot, 1st metatarsophalangeal joint, 2nd-5th metatarsophalangeal joint and hallux. A one-way ANOVA was used to test for significant differences in variables across the groups. Where differences existed Tukey post-hoc tests were used to ascertain the location of the difference.

**Results:**

Children who were obese and overweight demonstrated significantly (*p*<0.05) higher peak pressures and peak forces as well as significantly higher force-time and pressure–time integrals under the midfoot and 2nd-5th metatarsal regions. After normalisation of peak force, similar trends existed where the obese and overweight children demonstrated significantly (*p*<0.05) greater loading at the midfoot and 2nd-5th metatarsals.

**Conclusion:**

Findings from this study indicated that overweight children, as young as seven, displayed differences in foot loading during walking, when compared with normal weight children. These findings were consistent with loading patterns of children who were obese and suggest that early assessment and intervention may be required in overweight children to mitigate against the development of musculoskeletal complications associated with excessive body mass.

## Background

Childhood obesity is associated with multiple health co-morbidities [1] which predispose to increased health burden for children and economic burden for health service providers. Recent findings from the U.K. reported a combined prevalence of overweight and obesity to be 22.6% at 5 years of age, rising to 33.9% for children aged 11 years of age [2]. Current trends in obesity management are centred on weight loss and increased activity with a shift towards earlier intervention emerging.

Evaluation of foot loading is important to determine if obesity undermines the integrity of the foot as a weight bearing structure and as yet, conclusions on the impact of obesity on the paediatric foot are still lacking. Childhood obesity has been reported to be a risk factor for development of musculoskeletal pathologies affecting the lower limb [3,4] leading to altered walking characteristics [5-7], foot structure [8-10] and altered foot loading [11-14]. Despite this it is as yet undetermined whether overweight children display similar changes in foot loading to their obese counterparts. Further work is essential as early prevention and intervention will be key to mitigating against health co-morbidities associated with childhood obesity.

Utilising the current evidence to inform current care provision for children with obesity is challenging due to variations with the classification of obesity across studies, use of different pressure measurement technology, inconsistencies with protocols, differences in definition of foot segment(s) and lack of standardisation and reporting of plantar variables. The challenge with forming a consensus on the impact of obesity currently limits advances with care provision. The aim of this study was to determine foot loading characteristics in children aged 7 to 11 years, who were normal weight, overweight and obese, during level walking.

## Methods

### Participants

The sampling pool for this study consisted of one primary and two secondary schools based in East London, where pupils ranged from 4–11 years and 11–16 years. Male and female children aged 7 to 11 years were recruited for testing. Children were excluded from participation in the study if they disclosed a history of orthopaedic, neurological and/or musculoskeletal problems likely to affect their gait. Prior to recruitment, ethical approval for the study was granted from the University of East London Research Ethics Committee, London, UK (**ETH/08/94/0)**.

### Body mass index standard deviation score

Participants were classified according to their body mass index standard deviation score (BMI-SDS). A standard deviation or z-score expresses BMI in relation to normative data using population data from the 1990 British Growth Reference [15] and enables standardisation of data for age and gender [16]. Both mass and height were measured in accordance with standardised procedures [17] where height was measured to the nearest 0.1 cm using a portable, calibrated stadiometer and body mass to the nearest 0.05 kg using calibrated manual SECA body mass scales. Body mass index (BMI) was calculated as weight/height^2^. The body mass index SD scores (BMI-SDS) were derived using the following formula:

$$\mathrm{BMI}\;\mathrm{SDS}=\frac{\mathrm{BMI}\left(\mathrm{child}\right)‒\mathrm{Mean}\;\mathrm{BMI}\;\left(\mathrm{for}\;\mathrm{child}\;\mathrm{age}\right)}{1\;\mathrm{SD}\;\mathrm{of}\;\mathrm{BMI}\;\left(\mathrm{for}\;\mathrm{age}\right)}$$

This method has also been shown to be the optimal measure for assessing body mass on a single occasion in children [18]. Children with a BMI-SDS score greater than the 85th percentile were classified as overweight (BMI-SDS score ≥ 1.04) and greater than the 95th percentile were obese (BMI-SDS score ≥ 1.64) [19].

### Measurement apparatus

Dynamic plantar pressure data was collected with the MatScan® 3150 pressure distribution platform (TekScan, USA). This system consisted of a 5mm floor mat composed of 2288 resistive sensors, with a resolution of 1.4 sensors/cm^2^, a sensor matrix measuring 439.5 mm by 369.9 mm and a sampling frequency of 40 Hertz.

Gait velocity data was collected with SMARTSPEED photoelectric timing gates (Fusion Sport, AUS). This system uses polarised reflective photoelectric cells and records time to the nearest 1 millisecond.

### Plantar pressures

Protocols for the assessment of plantar pressures in children previously used in our laboratory were adhered to in this study [20]. All children were asked to walk barefoot along a 5-metre walkway with the MatScan® 3150 pressure distribution platform placed on a firm, level surface, in the centre of the walkway. All participants were given time to familiarise themselves with the process of walking over the platform to ensure they were comfortable with the procedure. Throughout data collection all participants were encouraged to adopt a natural gait pattern and to walk at a self-selected speed. Participants were asked to strike the platform with their fourth step to ensure a constant velocity had been reached prior to contact with the platform [21]. Trials were excluded and repeated if a participant appeared to target the platform and alter their gait pattern to ensure full contact with the mat, if the participant paused on the mat whilst walking, or if the participant did not continue to walk past the mat with at least five steps. Three complete trials of the right foot were recorded for each participant. To satisfy assumptions of data independence [22] data from the dominant foot was collected and this was the right foot for all participants.

**Figure 1:**
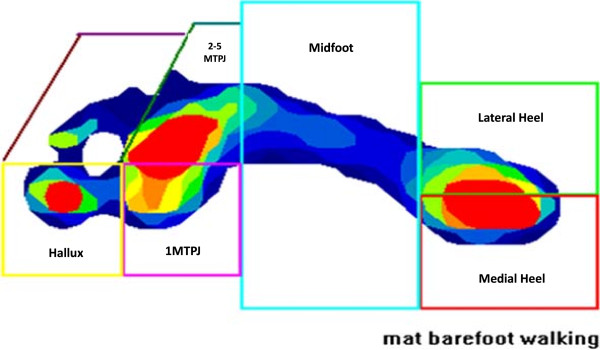
An example of a typical plantar pressure produced by the TekScan MatScan® system, displaying the six masked regions used during analysis.

Measures of peak pressure (kPa), peak force (N), peak force normalised to body mass (N/kg) and temporal measures of pressure–time integrals (kPa/s) and force-time integrals (N/s) were selected. Following data collection MatScan® Research Software version 6.4 was used to construct individual foot masks dividing the foot into six discrete regions: lateral heel, medial heel, midfoot, 1st metatarsophalangeal joint (1MTPJ), 2nd-5th metatarsophalangeal joint (2-5MTPJ) and hallux (Figure 1). All footprints were masked by the lead author and the same foot mask was applied across all trials for each participant. A single rater was chosen to perform all the manual masking to reduce variability, with previous research showing good reliability of manual mask application when performed by a single rater [23,24].

### Statistical analysis

Statistical analysis was conducted using SPSS version 15.0 for Windows (SPSS Inc., Chicago, USA). All data was tested for normality using the Kolmogoroff-Smirnov one-sample test. Means and standard deviations (SD) were calculated for each variable. A one-way Analysis of Variance (ANOVA) was used to test for significant differences in plantar loading variables across the groups (obese, overweight and normal weight). Where differences existed between groups Tukey post-hoc tests were used to ascertain the location of the difference between the three groups. The level of significance was set at *p*<0.05.

## Results

One hundred children were recruited into the study; participants were grouped according to BMI-SDS and there were three groups: normal weight, overweight and obese. Participant demographics, including gait velocity, are presented in Table 1. No significant differences were recorded in the gait velocity of the three groups. In all cases all variables demonstrated normal distribution.

**Table 1:** Anthropometric and gait velocity data [Mean (SD)] for the obese, overweight and normal weight children

	**Obese children (N=22)**	**Overweight children (N=22)**	**Normal weight children (N=56)**
Gender (M/F)	15/7	16/6	31/25
Age (years)	9.95 (1.56)	9.41 (1.74)	9.16 (1.56)
Body mass (kg)	55.75 (15.84)	39.91 (8.23)	30.59 (8.14)*†
Stature (m)	1.50 (0.14)	1.45 (0.12)	1.39 (0.10)*
Body mass index (kg/m^2^)	24.16 (3.14)	19.17 (1.28)	15.63 (2.04) *†
BMI-SDS	2.39 (0.49)	1.30 (0.15)	−0.62 (1.25) *†
Gait Velocity (m/sec)	1.06 (0.35)	1.10 (0.14)	1.12 (0.10)

* denotes a significant difference between obese and normal weight children (*p*<0.05).

† denotes a significant difference between overweight and normal weight children (*p*<0.05).

### Peak pressure and force

Descriptive data for peak pressure (kPa), peak force (N) and normalised peak force (N/kg) in the children who were obese, overweight and normal weight is summarised in Table 2. Significant group differences for peak pressure (*p*<0.05) and peak force (*p*<0.05) were located at the midfoot and 2nd-5th metatarsals between the overweight and normal weight children, where peak pressures and peak forces were higher for the overweight group. Significant group differences for peak pressure (*p*<0.05) at the lateral heel, midfoot and 2nd-5th metatarsals were found between the obese and normal weight children. Significant group differences for peak force (*p*<0.05) at the lateral heel, medial heel, midfoot and 2nd-5th metatarsals regions in comparison to the normal weight children were also found. All values were higher for the obese children.

**Table 2:** Summary of the dynamic peak pressure (kPa), peak force (N) and normalised force (N/kg) data for the obese (n=22), overweight (n=22) and normal weight children (n=56) [Mean (SD) of three trials]

**Mean of three trials**									
	**Peak pressure (kPa)**			**Peak force (N)**			**Normalised force (N/kg)**		
**Region**	**Obese**	**Overweight**	**Normal weight**	**Obese**	**Overweight**	**Normal weight**	**Obese**	**Overweight**	**Normal weight**
**Lateral heel**	289.26 (79.73)	249.85 (55.22)	221.78 (85.15)*	899.58 (217.29)	762.05 (191.68)	641.28 (168.98)*	1.64 (0.40)	1.95 (0.49)	2.04 (0.56)
**Medial heel**	269.02 (81.9)	236.11 (54.53)	220.08 (92.56)	782.81 (232.05)	655.43 (122.75)	595.24 (242.30)*	1.43 (0.42)	1.67 (0.31)	1.98 (0.81)
**Midfoot**	110.88 (47.92)	95.23 (52.75)	58.33 (38.97)*†	367.16 (121.76)	318.72 (138.65)	173.59 (85.01)*†	0.67 (0.22)	0.65 (0.25)	0.48 (0.28)*†
**1MTPJ**	178.01 (19.94)	185.39 (82.03)	194.41 (59.82)	511.46 (208.44)	507.69 (184.64)	515.21 (135.82)	0.96 (0.38)	1.17 (0.47)	1.22 (0.45)
**2-5MTPJ**	277.49 (91.08)	244.85 (80.79)	198.41 (54.55)*†	1034.98 (358.7)	946.38 (253.83)	573.16 (143.37)*†	2.18 (0.47)	2.16 (0.65)	1.81 (0.48)*†
**Hallux**	211.01 (19.94)	214.19 (79.77)	216.47 (86.08)	673.72 (197.07)	635.72 (206.8)	696.36 (178.51)	0.92 (0.38)	1.09 (0.50)	2.32 (0.59)

* denotes a significant difference between obese and normal weight children (*p*<0.05).

† denotes a significant difference between overweight and normal weight children (*p*<0.05).

Differences in peak force data were largely eliminated once normalised to body mass. However the results at the midfoot and 2nd-5th metatarsals remained, with the children who were overweight and obese demonstrating increased loading at these sites. Post-hoc analysis revealed significant group differences (*p*<0.05) between the overweight and obese participants in comparison to the normal weight children at both these regions of the foot.

### Pressure–time and force-time integrals

Temporal characteristics of foot loading (pressure–time integrals and force-time integrals) for the groups are summarised in Table 3. Elevated pressure–time and force-time integrals for the overweight and obese children were found. Significant group differences (*p*<0.05) at the midfoot and 2nd-5th metatarsals between children who were overweight and normal weight were found. Significant group differences (*p*<0.05) were also found at the lateral heel and medial heel, midfoot and 2nd-5th metatarsals when comparing children who were obese and normal weight participants.

**Table 3:** Summary of the pressure–time integral (kPa/s) and force-time integral (N/s) data for the obese (n=22), overweight (n=22) and normal weight children (n=56) [Mean (SD) of three trials]

**Mean of three trials**						
	**Pressure–time integral (kPa/s)**			**Force-time integral (N/s)**		
**Region**	**Obese**	**Overweight**	**Normal weight**	**Obese**	**Overweight**	**Normal weight**
**Lateral heel**	46.90 (22.47)	35.26 (11.64)	29.14 (11.49)*	743.46 (379.16)	426.80 (169.88)	388.24 (94.42)*
**Medial heel**	46.59 (26.12)	34.84 (12.19)	29.48 (10.57)*	615.32 (360.77)	364.62 (142.79)	243.76 (57.94)*
**Midfoot**	21.80 (8.71)	18.93 (9.82)	10.02 (6.22)*†	594.88 (322.18)	432.58 (267.47)	115.07 (97.33)*†
**1MTPJ**	38.70 (14.96)	32.09 (13.09)	40.89 (17.56)	220.29 (185.41)	262.62 (175.47)	277.55 (147.37)
**2-5MTPJ**	46.74 (15.26)	44.33 (16.42)	33.83 (9.47)*†	994.41 (667.49)	625.98 (431.39)	460.40 (153.04)*†
**Hallux**	31.41 (31.42)	30.75 (13.47)	32.18 (19.64)	163.71 (131.67)	172.94 (135.34)	234.97 (134.36)

* denotes a significant difference between obese and normal weight children (*p*<0.05).

† denotes a significant difference between overweight and normal weight children (*p*<0.05).

## Discussion

The aim of this study was to determine foot loading characteristics in healthy weight, overweight and obese children aged 7 to 11 years during level walking. Our findings identified that that children aged 7 to 11 years, who were overweight, displayed marked differences in foot loading, when compared with normal weight children. Overweight children generated significantly greater peak pressures and peak forces at the midfoot and 2nd-5th metatarsal regions of the foot, a trend that was similar to the obese children. Overweight children also generated increased force-time and pressure–time integrals under the midfoot and 2nd-5th metatarsals. A similar trend was found for the obese children with significant differences also found at the lateral and medial heel in comparison to the normal weight children.

Significant differences in the loading characteristics of overweight and obese children in this present study are consistent with previous research [11-14]. Previous findings have identified elevated levels of loading, in children who are obese, at the plantar heel, midfoot and 2nd-5th metatarsals when compared to non-obese children [11-14]. Previous studies have limited comparisons to children who are obese and normal weight and the findings from our study demonstrated that children who are overweight display similar foot loading characteristics to children who are obese, with significantly increased peak and temporal loading at the midfoot and 2nd-5th metatarsals. These findings support the view that children who are overweight and obese could be at risk of developing foot discomfort as a consequence of the increased pressures and forces acting upon the immature musculoskeletal structure of the paediatric foot.

Elevated and prolonged patterns of foot loading at the midfoot in both overweight and obese groups suggest increased ground contact of the midfoot during walking. This could be a result of excessive adipose tissue around the medial foot leading to increased loading, although it has been demonstrated that obese children do not have greater adipose tissue than non-obese children [25]. Additionally, recent work has reported low correlations between medial midfoot plantar pressures and midfoot plantar fat pad thickness [26]. An alternative suggestion for increased loading at these sites may be a change in the biomechanical function of the foot. There is a common view that obese children have a pes planus foot posture and the loading patterns demonstrated in this study were consistent with previous literature [8,10,12,14,25]. Song-hua and colleagues [14] citing the work of D’Aout et al. [27] stated that the foot is pronating when the loading (force and pressure) is higher under the medial region as demonstrated by both children who were overweight and obese in the present study. The findings were significant for the children who were obese compared to the normal weight children but not for the children who were overweight, although the trend in the data suggested an increase.

When peak force was normalised to body mass a significant increase at the midfoot and 2nd-5th metatarsals in the obese and overweight children was found. Thus, when normalised to body mass the obese and overweight participants still demonstrated a significant change in loading at these sites. This reiterates the suggestion that there is an underlying difference in the biomechanics of foot loading which indicated an elevated level of loading applied to the soft-tissue and joint structures. These findings are consistent with the view that children who are obese are at an increased risk of developing foot discomfort and or pathology [3,4], as a consequence of the increased pressures and forces acting upon the immature musculoskeletal structure of the paediatric foot. Interestingly, no significant group differences were reported for peak pressure, peak and normalised force, pressure–time integral and force-time integral values at the hallux. The trend is our data suggested a decrease in loading which may further support the view that overweight and obesity affected the biomechanical function of the foot. Further work is required to explore the association between foot function and pathology in overweight and obese children and the potential for intervention to encourage a more medial loading pattern to alleviate increased pressures and forces at the midfoot and 2nd-5th metatarsals.

Emerging from this study is the view that earlier intervention and consideration of the foot in children who are overweight may be required to mitigate against further health co-morbidities. Previous research has indicated that children who generated higher peak loading characteristics on the plantar surface of the foot were more likely to suffer pain and discomfort [28]. Foot discomfort and/or pain associated with increased tissue stress may have additional complications in-so-much that it may hinder their participation in physical activity as weight bearing activities can become difficult if not appropriately designed to account for these functional changes [28]. Whether the increased pressures and forces, as demonstrated in this study, were associated with foot discomfort to deter the obese and overweight children from participating in physical activity, and thereby perpetuating the cycle of obesity and exacerbating foot problems, require further investigation.

There are several limitations to this present study. The spatial resolution of the system was low (1.4 sensors/cm^2^) which may affect the isolation of small regions of the foot and accuracy of the data. Furthermore, we did not control velocity of walking as previous work with children demonstrated increased gait asymmetry when precise gait instructions were given [29,30]. Further longitudinal research is required to advance our understanding into the impact of obesity on the structural development and function of the foot in children across a broader age range; with a particular focus on investigating the age at which structural and functional changes to the foot (as a result of increased adiposity) become apparent. Finally, the specific causative factors associated with obesity and functional foot changes in children cannot be confirmed from this work. It would be beneficial for future research to consider dynamic function of the paediatric foot and the impact of this. Particular attention should be given to establishing relationships between increased body mass, biomechanical function and the development of musculoskeletal pathology.

The present study has demonstrated differences in the plantar foot loading characteristics of obese, overweight and normal weight children, aged 7–11 years, during level walking. Significant findings were identified as increases in peak and temporal characteristics of foot loading in obese and overweight children at the midfoot and 2nd-5th metatarsals in comparison to the normal weight children, suggesting that these areas of the foot may be exposed to a higher level of loading being applied to the soft-tissue and joint structures and, in turn may be vulnerable to foot pain, discomfort and injury in young children. These results also indicated that changes in a child’s foot loading occur earlier than anticipated and may be associated with a smaller increase in body mass than first thought.

## Conclusion

Advancing our understanding of the impact of overweight on the foot and lower limb is important to inform clinical management and ensure that multi-disciplinary care is appropriately targeted. The present study has demonstrated differences in foot loading characteristics in young overweight children. Changes to foot loading occurred earlier than previously documented and this finding reinforces the view that early intervention is required to mitigate against the impact of obesity on the foot and lower limb. As the long-term consequences of altered foot loading profiles are currently unknown, there is a need for further research to explore the short- and long-term impacts of increased body mass on the structural and functional characteristics of children’s feet during walking.

## Consent

Written informed consent was obtained from the parent/guardian and written assent obtained from each participant prior to recruitment.

## Abbreviations

1MTPJ: 1st metatarsophalangeal joint; 2-5MTPJ: 2nd-5th metatarsophalangeal joint.

## Competing interests

None of the authors have any financial or personal relationships with other people or organisations that could inappropriately influence this work.

## Authors’ contributions

SDC, SCM and WID all conceived and designed the study. SDC collected and analysed the data. SDC drafted the manuscript with the assistance of both SCM and WID. All three authors approved the final manuscript.
